# A prospective randomized study comparing effects of empagliflozin to sitagliptin on cardiac fat accumulation, cardiac function, and cardiac metabolism in patients with early-stage type 2 diabetes: the ASSET study

**DOI:** 10.1186/s12933-021-01228-3

**Published:** 2021-02-02

**Authors:** Shigenori Hiruma, Fumika Shigiyama, Shinji Hisatake, Sunao Mizumura, Nobuyuki Shiraga, Masaaki Hori, Takanori Ikeda, Takahisa Hirose, Naoki Kumashiro

**Affiliations:** 1 Division of Diabetes, Metabolism and Endocrinology, Department of Medicine, Toho University Graduate School of Medicine, 6-11-1 Omori-Nishi, Ota-ku, Tokyo, Japan; 2 Division of Cardiovascular Medicine, Department of Internal Medicine, Toho University Graduate School of Medicine, 6-11-1 Omori-Nishi, Ota-ku, Tokyo, Japan; 3 Department of Radiology, Toho University Omori Medical Center, 6-11-1 Omori-Nishi, Ota-ku, Tokyo, Japan

**Keywords:** DPP-4 inhibitor, Early-stage type 2 diabetes mellitus, Epicardial fat, ^123^I-BMIPP scintigraphy, Myocardial triglyceride content, Pericardial fat, Preserved cardiac function, SGLT2 inhibitor

## Abstract

**Background:**

While the cardioprotective benefits of sodium-glucose cotransporter-2 (SGLT2) inhibitors have been established in patients with cardiovascular disease (CVD), their advantages over other anti-diabetic drugs at earlier stages remain unclear. We compared the cardioprotective effects of empagliflozin, an SGLT2 inhibitor, with those of sitagliptin, a dipeptidyl peptidase-4 (DPP-4) inhibitor, focusing on cardiac fat accumulation, cardiac function, and cardiac metabolism in patients with early-stage type 2 diabetes mellitus (T2DM) without CVD complications.

**Methods:**

This was a prospective, randomized, open-label, blinded-endpoint, parallel-group trial that enrolled 44 Japanese patients with T2DM. The patients were randomized for 12-week administration of empagliflozin or sitagliptin. Pericardial fat accumulation and myocardial triglyceride content were evaluated by magnetic resonance imaging and proton magnetic resonance spectroscopy, respectively. Echocardiography, ^123^I-β-methyl-iodophenyl pentadecanoic acid myocardial scintigraphy, and laboratory tests were performed at baseline and after the 12-week treatment period.

**Results:**

The patients were middle-aged (50.3 ± 10.7 years, mean ± standard deviation) and overweight (body mass index 29.3 ± 4.9 kg/m^2^). They had a short diabetes duration (3.5 ± 3.2 years), HbA1c levels of 7.1 ± 0.8%, and preserved cardiac function (ejection fraction 73.8 ± 5.0%) with no vascular complications, except for one baseline case each of diabetic nephropathy and peripheral arterial disease. After the 12-week treatment, no differences from baseline were observed between the two groups regarding changes in pericardial, epicardial, and paracardial fat content; myocardial triglyceride content; cardiac function and mass; and cardiac fatty acid metabolism. However, considering cardiometabolic biomarkers, high-density lipoprotein cholesterol and ketone bodies, including β-hydroxybutyric acid, were significantly increased, whereas uric acid, plasma glucose, plasma insulin, and homeostasis model assessment of insulin resistance were significantly lower in the empagliflozin group than in the sitagliptin group (p < 0.05).

**Conclusions:**

Although the effects on cardiac fat and function were not statistically different between the two groups, empagliflozin exhibited superior effects on cardiometabolic biomarkers, such as uric acid, high-density lipoprotein cholesterol, ketone bodies, and insulin sensitivity. Therefore, when considering the primary preventive strategies for CVD, early supplementation with SGLT2 inhibitors may be more beneficial than DPP-4 inhibitors, even in patients with early-stage T2DM without current CVD complications.

*Clinical Trial Registration:* UMIN000026340; registered on February 28, 2017. https://upload.umin.ac.jp/cgi-open-bin/icdr_e/ctr_view.cgi?recptno=R000030257

## Background

Type 2 diabetes mellitus (T2DM) is a major risk factor for cardiovascular disease (CVD) [1, 2]. Various glucose-lowering agents, including sodium-glucose cotransporter-2 (SGLT2) inhibitors and dipeptidyl peptidase-4 (DPP-4) inhibitors, are commonly administered for the treatment of T2DM. The former lowers blood glucose levels by suppressing reuptake of sodium and glucose from primitive urine and has been shown to reduce CVD events in patients with/without T2DM, as well as in those at high risk of CVD or those with reduced cardiac function [3–6]. The latter lowers blood glucose levels by increasing incretin hormone levels but does not reduce CVD events [7–10]. Hence, a simple reduction in blood glucose levels is not sufficient for cardiovascular protection, and other mechanisms are involved.

It has been suggested that cardiac fat accumulation causes excessive release of proinflammatory cytokines and free fatty acids, resulting in myocardial intracellular lipotoxicity, myocardial fibrosis, and cardiac dysfunction [11–17]. Although both SGLT2 inhibitors and DPP-4 inhibitors reduce epicardial fat [18–22], no clinical studies have been performed to compare the effectiveness of these inhibitors on pericardial and epicardial fat, as well as myocardial triglyceride content. However, Lee et al. recently reported that SGLT2 inhibitors (empagliflozin and dapagliflozin) were superior to DPP-4 inhibitors for improving cardiac function after 24 months of treatment in patients with T2DM, as well as in those with reduced cardiac function and previous CVD events [23]. Moreover, regarding cardiac metabolism, it is well-known that the impaired heart switches energy sources from fatty acids to glucose [24]. Hence, disordered cardiac fatty acid metabolism could be associated with impaired myocardial lipolysis, increased myocardial triglyceride content, abnormal cardiac wall motion, and future cardiac events [25–28]. Recently, SGLT2 inhibitors were reported to induce a global change in energy substrates from glucose to lipids throughout the body [29]. However, the effects of SGLT2 inhibitors and DPP-4 inhibitors on cardiac fatty acid metabolism remain unknown.

Accumulating evidence suggests the superiority of SGLT2 inhibitors over DPP-4 inhibitors in their cardioprotective role in patients with past CVD events or high CVD risks [3–10, 23, 30–33]. However, it remains unclear whether SGLT2 inhibitors are superior to DPP-4 inhibitors in reducing cardiovascular or cardiometabolic risk factors in patients with early-stage T2DM, without CVD, and with preserved cardiac function.

In this study, we hypothesized that SGLT2 inhibitors elicit more cardioprotective effects than DPP-4 inhibitors, specifically in patients with early-stage T2DM and without CVD (including not having heart failure). Thus, considering the primary prevention of CVD, this study was performed to compare the effects of empagliflozin and sitagliptin on CVD risk factors, including pericardial and epicardial fat accumulation, myocardial triglyceride content, cardiac function, cardiac fatty acid metabolism, and metabolic biomarkers in patients with early-stage T2DM, without a history of CVD events.

## Methods

### Study design

This was a prospective, randomized open-label, blinded-endpoint study. The design and rationale have been reported previously [34]. This study was registered in the University Hospital Medical Information Network Clinical Trial Registry (UMIN000026340), a nonprofit organization in Japan that meets the requirements of the International Committee of Medical Journal Editors, and it was approved by the certified clinical research review board of Toho University (CRB3180016), as well as the Ethics Committee of Toho University Omori Medical Center (M16193). This study was conducted according to the Declaration of Helsinki and current legal regulations in Japan. To avoid bias in the collected data, the processes of enrollment, randomization, data management, and analysis were conducted by a third-party (Soiken, Inc., Tokyo, Japan).

### Study population

The target number of patients required for registration was 44. Recruitment for the study began in April 2017 and ended in March 2019 at the Toho University Omori Medical Center. The inclusion criteria were as follows: (1) T2DM patients with proper diet and exercise therapy alone or prescribed α-glucosidase inhibitors, sulfonylureas, glinides, or combinations of these agents; (2) patients with glycated hemoglobin (HbA1c) levels of 6.0–10.0%; (3) patients aged 20–74 years; (4) patients with a body mass index of ≥ 22 kg/m^2^; and (5) patients who provided written informed consent. The exclusion criteria were: (1) patients with type 1 diabetes mellitus or secondary diabetes mellitus; (2) patients with renal dysfunction (estimated glomerular filtration rate < 45 mL/min/1.73 m^2^); (3) patients with a medical history of cerebral infarction or stroke within 12 weeks prior to giving consent for enrollment; (4) patients with a past medical history of myocardial infarction, angina pectoris, or present medical history of atrial fibrillation; (5) patients with left ventricular ejection fraction (LVEF) < 30%; (6) patients with infection; (7) patients with untreated cancer; (8) patients with collagen diseases, with the exception of well-controlled disease progression with prednisolone ≤ 5 mg/day; (9) patients with hepatic cirrhosis; (10) patients with liver failure that was virus-, autoimmune- or drug-induced; (11) patients with alcoholism; (12) pregnant or breastfeeding patients, or those planning to become pregnant during the course of the study; (13) patients allergic to empagliflozin or sitagliptin; and (14) patients with anemia (hemoglobin < 12 g/dL).

### Randomization and study intervention

After consent and enrollment, baseline checkups were performed for each subject. Within 2 weeks of checkup, eligible subjects were randomly and equally assigned to the empagliflozin add-on group (empagliflozin 10 mg/day) or sitagliptin add-on group (sitagliptin 50 mg/day as the initial dose). Randomization was performed by a computer-based dynamic allocation method using the presence of sulfonylurea administration and intrahepatic lipid content assessed by proton magnetic resonance spectroscopy (^1^H-MRS) at baseline as the assignment factors. After treatment initiation, vital signs were assessed and urinalysis and blood tests were performed to screen for the occurrence of adverse events at 4 weeks; concurrently, the dosage of sitagliptin was increased to 100 mg/day. After the 12-week treatment period, the same assessments were conducted as those performed at baseline.

### Study outcomes

The primary endpoint of this study was the change in amount of pericardial fat, accounting for the sum of epicardial and paracardial fat, as evaluated by magnetic resonance imaging (MRI). The secondary endpoints were the changes from baseline to 12 weeks in the following parameters: (1) myocardial triglyceride content measured by ^1^H-MRS; (2) cardiac function and mass estimated by echocardiography; (3) indicators of cardiac fatty acid metabolism assessed by iodine-123-β-methyl-iodophenyl pentadecanoic acid myocardial scintigraphy (^123^I-BMIPP scintigraphy); (4) blood biomarkers (see Table 4 and Additional file 1); (5) body weight and blood pressure; (6) medication compliance; and (7) incidence of adverse events (AEs).

### Magnetic resonance imaging (MRI)

Pericardial fat accumulation was estimated by cine MRI with a 1.5 T whole-body MR scanner (MAGNETOM AvantoSQ1.5T B-19; Siemens, Erlangen, Germany) [35, 36]. Four-chamber cine sequences were obtained using a steady-state free precession sequence. Several cine images were used to generate a complete view of the left ventricle (LV) from the base to the apex. Scanning was performed with typical imaging parameters: repetition time, 68.4 ms; echo time, 1.48 ms; flip angle, 80 degrees; matrix, 134 × 192; field of view, 360 × 360 mm; slice thickness, 10 mm; gap, 0 mm; and calculated phases, 25. Epicardial fat and paracardial fat were estimated by a non-participating doctor using dedicated software (SYNAPSE VINCENT, Fujifilm Corporation, Tokyo, Japan). The high signal range between the myocardium and pericardium was designated as the epicardial fat area. Similarly, the high signal range outside the pericardium was considered the paracardial fat area. The pericardial fat area was calculated as the sum of epicardial and paracardial fat areas.

### Proton magnetic resonance spectroscopy (^1^H-MRS)

The myocardial triglyceride content was measured by ^1^H-MRS analysis using a 1.5 T whole-body MR scanner (MAGNETOM AvantoSQ 1.5 T B-19) performed by specialists with dedicated software (Argus; Siemens), as described previously [36–38]. The volume of interest (VOI = 10 × 10 × 20 mm^3^) was manually placed on the ventricular septum of the cine images of the heart and adjusted to fit the ventricular septum of the LV. The spectra of lipid and water were acquired using point-resolved spectroscopy sequences (repetition time/echo time, 4000/30 ms). The myocardial signal was quantified as the triglyceride signal intensity at 1.4 ppm from the spectra with water suppression, and the water signals were quantified at 4.7 ppm from the spectra without water suppression.

### Echocardiography

Echocardiography was performed by experienced technicians with 3.5- and 2.5-MHz transducers for two-dimensional, M-mode, and continuous-wave Doppler measurements. The percent fractional shortening of the ventricle (%FS) was calculated as follows:  %FS = {(left ventricular end-diastolic dimension-left ventricular end-systolic dimension)/left ventricular end-diastolic dimension} × 100.

### Iodine-123-β-methyl-iodophenyl pentadecanoic acid myocardial scintigraphy (^123^I-BMIPP scintigraphy)

Cardiac fatty acid metabolism was assessed by ^123^I-BMIPP scintigraphy using 111 MBq of an ^123^I-BMIPP radiotracer (111 MBq/1.5 mg, Nihon Medi-Physics Co., Ltd., Tokyo, Japan). Early images were captured 20 min after injection of the radiotracer, and delay images were captured at 3 h, both with a dual-headed single-photon emission computed tomography (SPECT) gamma camera (Infinia H3000WT; GE Medical System, Tel Aviv, Israel). The SPECT data were acquired in step shooting mode using two detectors (180° rotation) at a matrix size of 64 × 64. A series of contiguous transaxial images with 5.89 mm thickness were reconstructed using the Butterworth filtered back-projection algorithm (order, 10; cut-off, 0.40 cycles/cm) without attenuation or correction. Regional tracer uptake was scored semi-quantitatively from 0 (normal) to 4 (severe defect) for 17 segments of the LV, and the sum of the defect scores for all segments was calculated to derive the summed rest score (SRS) [28]. The heart washout rate between 20 min and 3 h after intravenous injection of ^123^I-BMIPP was calculated as follows: [(count at 20 min − count at 3 h)/count at 20 min] × 100. The count per pixel data was measured for both the heart and mediastinum, and the ratio of heart-to-mediastinal uptake at 20 min and 3 h after injection of ^123^I-BMIPP was calculated [39].

### Laboratory testing

Blood and urine samples were collected at Toho University Hospital and submitted to the central laboratory of the hospital or a private laboratory (SRL laboratory, Tokyo, Japan). All data were collected after overnight fasting. Administration of any oral hypoglycemic agents, including empagliflozin and sitagliptin, was prohibited on the sampling day. Homeostasis model assessment-insulin resistance (HOMA-IR) was calculated as follows: HOMA-IR = (plasma insulin × plasma glucose)/405.

### Safety evaluation

During this study, the investigators continuously monitored any AEs through regular medical checkups. All related AEs, as well as side effects of the drug, abnormal values from the clinical tests, and unusual complaints, were reported and documented.

### Sample size calculation

No previous studies have compared the effects of empagliflozin and sitagliptin on cardiac fat accumulation. In addition, while planning this study, no studies were available for estimating the effect of the SGLT2 inhibitor and DPP-4 inhibitor on pericardial fat accumulation. Therefore, since hepatic steatosis was associated with cardiac lipid accumulation and cardiac dysfunction [36, 40–42], we referred to previous studies that demonstrated a reduction in the intrahepatic lipid content with sitagliptin administration [43, 44]. Based on these studies, we estimated that 16 subjects per group was sufficient for this study, and assuming a dropout rate of 25%, the target number of patients for enrollment was set as 22 per group.

### Statistical analysis

Analyses of the primary and secondary endpoints were performed on the full analysis set (FAS). The FAS includes all research subjects enrolled in this study and assigned to a study treatment. Subjects without primary endpoint data, or those who significantly violated the study protocol, were excluded. Safety analysis with AEs was performed on the safety analysis set, which included all subjects enrolled in this study who were administered all or part of the study treatment. We analyzed the primary endpoint using covariance models, including the baseline intrahepatic lipid content and presence of sulfonylurea medication as assignment factors. To compare categorical variables in the two groups, the Chi-square test was used for nominal variables, and a two-sample t*-*test was used for continuous variables, as all data were normally distributed. Statistical significance was defined as p < 0.05. All statistical analyses were performed independently by the administrative office of the ASSET study using SAS software version 9.4 (SAS Institute, Cary, NC, USA).

## Results

### Clinical characteristics

**Fig. 1:**
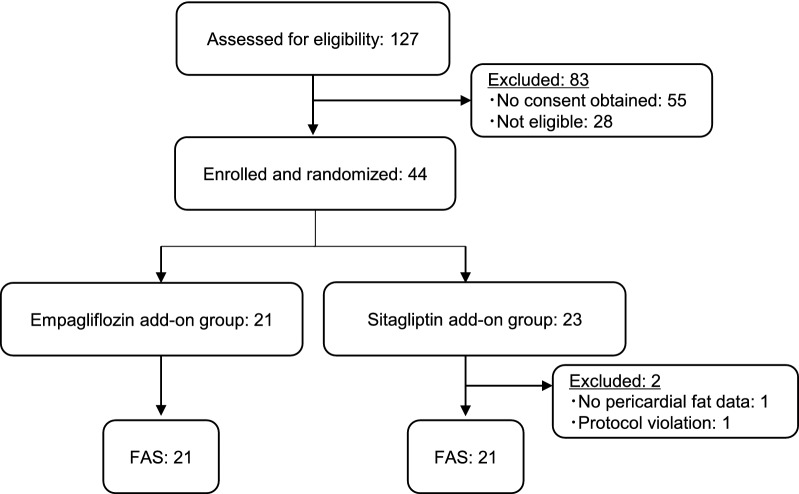
Flow diagram depicting phases of the parallel randomized trial for empagliflozin and sitagliptin groups. FAS: full analysis set

Figure 1 shows the flow diagram of the progress during the phases of this parallel randomized trial. A total of 127 subjects were screened and 83 were ineligible (55 subjects denied consent and 28 did not meet the inclusion criteria). Forty-four subjects were enrolled and randomized. Ultimately, 42 completed the study and were assigned to the FAS. Their baseline clinical characteristics are summarized in Table 1. Most patients were middle-aged (average, 50.3 years old) and overweight (average body mass index, 29.3 kg/m^2^), and their average HbA1c was 7.1%. The duration of diabetes was short (average 3.5 years), and 19 subjects were drug-naïve before enrollment. Two subjects had microvascular/macrovascular complications (one subject had diabetic nephropathy and another had peripheral arterial disease). There were no differences in any baseline clinical characteristics between the two groups. Although it was recommended to increase the sitagliptin dose to 100 mg/day during the trial, two patients remained at 50 mg/day throughout the study period at the doctor’s discretion.

**Table 1:** Baseline characteristics of patients in empagliflozin group and sitagliptin group

	Empagliflozin	Sitagliptin	p value
Age (years)	52.8 ± 9.7	47.8 ± 11.5	0.140
Sex (males/females), n (%)	16 (76.2)/5 (23.8)	15 (71.4)/6 (28.6)	0.726
Duration of diabetes (years)	3.9 ± 3.7	3.0 ± 2.7	0.403
Body weight (kg)	80.3 ± 19.0	84.4 ± 16.1	0.469
Body mass index (kg/m^2^)	28.6 ± 4.8	30.0 ± 5.0	0.398
HbA1c (%)	7.1 ± 0.8	7.0 ± 0.9	0.684
eGFR (mL/min/1.73 m^2^)	86.5 ± 19.0	87.1 ± 14.4	0.907
Fasting plasma insulin (μU/mL)	13.7 ± 7.1	14.4 ± 9.5	0.795
Free fatty acid (μEq/L)	710.2 ± 164.2	689.8 ± 209.0	0.727
BNP (pg/mL)	8.9 ± 5.8	13.5 ± 12.8	0.138
Pericardial fat (mm^2^)	2310.1 ± 1065.1	2462.0 ± 947.7	0.628
Myocardial triglyceride content (%)	5.9 ± 9.8	3.1 ± 3.1	0.220
Microvascular complications, n (%)			
Diabetic retinopathy	0 (0.0)	0 (0.0)	–
Diabetic nephropathy	1 (4.8)	0 (0.0)	1.000
Diabetic neuropathy	0 (0.0)	0 (0.0)	–
Macrovascular complications, n (%)			
Cerebrovascular disease	0 (0.0)	0 (0.0)	–
Coronary disease	0 (0.0)	0 (0.0)	–
Peripheral arterial disease	1 (4.8)	0 (0.0)	1.000
Anti-diabetic drugs, n (%)			
α-Glucosidase inhibitors	5 (23.8)	5 (23.8)	1.000
Glinides	4 (19.0)	3 (14.3)	1.000
Sulfonylureas	7 (33.3)	7 (33.3)	1.000
Antihypertensive drugs, n (%)			
Diuretic drugs	1 (4.8)	2 (9.5)	1.000
Calcium channel blockers	6 (28.6)	3 (14.3)	0.454
Angiotensin-converting enzyme inhibitors	0 (0.0)	0 (0.0)	–
Angiotensin II receptor blockers	7 (33.3)	2 (9.5)	0.130
α-Blockers	0 (0.0)	0 (0.0)	–
β-blockers	1 (4.8)	0 (0.0)	1.000

Data are presented as the mean ± SD or n (%); n = 21 for both groups. p values < 0.05 indicate significant differences. Comparisons were performed using the Chi-square test for categorical variables and two-sample *t*-tests for continuous variables. BNP: brain natriuretic peptide, eGFR: estimated glomerular filtration rate

### Cardiac fat accumulation

**Fig. 2:**
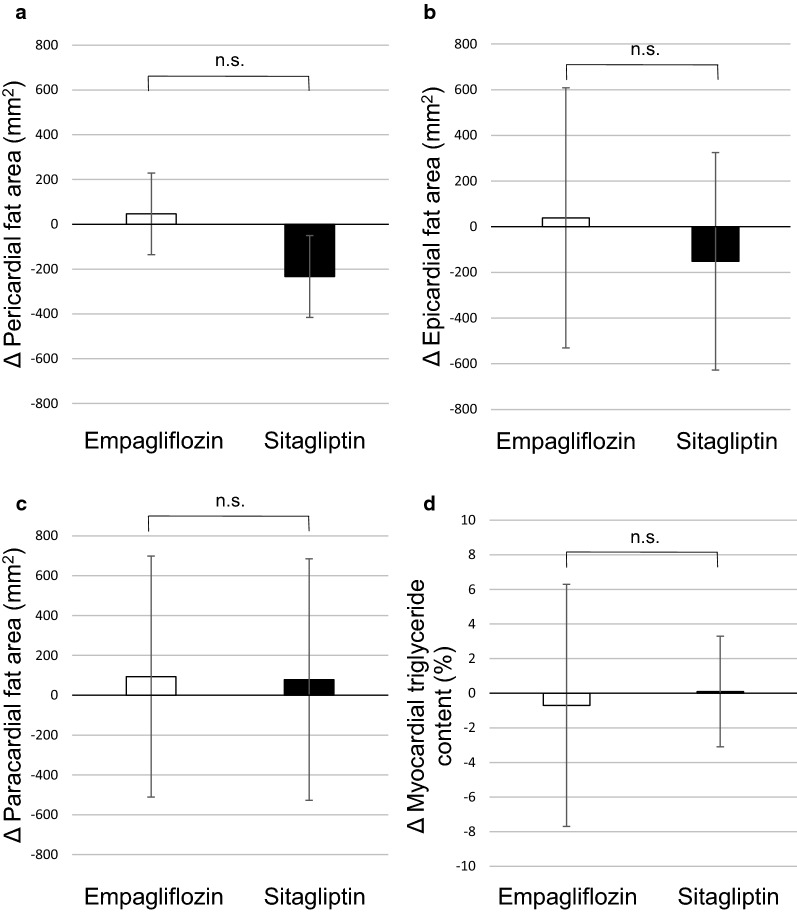
Change in cardiac fat accumulation compared to baseline values. **a** Pericardial fat accumulation, **b** epicardial fat accumulation, **c** paracardial fat accumulation, and **d** myocardial triglyceride content between empagliflozin and sitagliptin groups. Comparisons were performed by two-sample *t*-tests. n.s., not significant

There was no difference in the change in accumulation of pericardial fat, which is composed of epicardial and paracardial fat, between the two groups (46.8 ± 182.4 vs. −33.0 ± 182.4 mm^2^, empagliflozin group vs. sitagliptin group, respectively, p = 0.27, Fig. 2a–c). The change in myocardial triglyceride content was also not statistically different between the groups (−0.7 ± 7.0% vs. 0.1 ± 3.2%, p = 0.64, Fig. 2d).

### Cardiac function and mass assessed by echocardiography

Table 2 shows the echocardiography parameters at baseline and after 12 weeks, as well as the change in each parameter. No significant differences were observed in the changes of each parameter between the two groups. However, LVEF and %FS were significantly decreased only in the sitagliptin group over the 12-week study period (ΔLVEF: −1.6 ± 3.0%, Δ %FS: −1.4 ± 2.7%, both p < 0.05).

**Table 2:** Echocardiography parameters

	Empagliflozin	Sitagliptin	p value
LVEF (%)			
Baseline	73.4 ± 5.6	74.2 ± 4.5	0.620
12 weeks	73.5 ± 4.2	72.6 ± 5.0	0.539
Change	0.1 ± 4.0	−1.6 ± 3.0	0.138
Intragroup p value	0.932	0.026*	
%FS (%)			
Baseline	42.9 ± 4.6	43.5 ± 4.0	0.625
12 weeks	42.9 ± 3.5	42.1 ± 4.1	0.521
Change	0.0 ± 3.5	−1.4 ± 2.7	0.147
Intragroup p value	0.985	0.024*	
E/e′			
Baseline	10.9 ± 3.6	10.0 ± 2.7	0.397
12 weeks	10.3 ± 3.2	9.3 ± 1.8	0.188
Change	−0.5 ± 2.4	−0.8 ± 3.1	0.780
Intragroup p value	0.310	0.256	
E/A			
Baseline	0.88 ± 0.25	1.01 ± 0.31	0.132
12 weeks	0.92 ± 0.23	1.01 ± 0.36	0.307
Change	0.04 ± 0.17	0.00 ± 0.19	0.500
Intragroup p value	0.296	0.963	
Cardiac index (L/min/m^2^)			
Baseline	3.32 ± 0.75	3.14 ± 0.60	0.408
12 weeks	3.41 ± 0.60	3.03 ± 0.55	0.036*
Change	0.09 ± 0.74	−0.12 ± 0.53	0.296
Intragroup p value	0.566	0.325	
LV mass (g)			
Baseline	153.6 ± 43.0	161.1 ± 38.2	0.555
12 weeks	152.1 ± 44.4	155.1 ± 32.5	0.804
Change	−1.5 ± 15.9	−6.0 ± 19.7	0.424
Intragroup p value	0.666	0.180	

Data are presented as the mean ± SD (n = 21 for both groups). p values < 0.05 indicate significant differences. Comparisons were performed by one-sample *t*-test in each group, and two-sample *t*-tests between groups. LVEF: left ventricle ejection fraction, FS: fractional shortening, E/e′: ratio of mitral peak velocity of early filling to early diastolic mitral annular velocity, E/A: early filling/atrial filling ratio. *p < 0.05

### Parameters of cardiac fatty acid metabolism assessed by ^123^I-BMIPP scintigraphy

No differences were observed between the groups in any parameters of the ^123^I-BMIPP scintigraphy (Table 3). However, SRS was significantly decreased from baseline during the 12 weeks in the sitagliptin group (ΔSRS: −0.35 ± 0.67, p < 0.05).

**Table 3:** Parameters of iodine-123-β-methyl-iodophenyl pentadecanoic acid myocardial scintigraphy

	Empagliflozin	Sitagliptin	p value
SRS			
Baseline	0.43 ± 0.68	0.80 ± 1.82	0.388
12 weeks	0.19 ± 0.40	0.43 ± 1.54	0.496
Change	−0.24 ± 0.89	−0.35 ± 0.67	0.653
Intragroup p value	0.234	0.031*	
Washout rate (%)			
Baseline	11.6 ± 3.9	11.3 ± 4.9	0.858
12 weeks	10.7 ± 3.7	12.1 ± 4.1	0.238
Change	−0.9 ± 4.3	0.7 ± 3.7	0.200
Intragroup p Value	0.354	0.379	
Early H/M ratio			
Baseline	2.65 ± 0.36	2.57 ± 0.31	0.496
12 weeks	2.58 ± 0.39	2.56 ± 0.36	0.879
Change	−0.07 ± 0.24	−0.01 ± 0.23	0.460
Intragroup p value	0.206	0.806	
Delay H/M ratio			
Baseline	2.36 ± 0.33	2.32 ± 0.34	0.725
12 weeks	2.34 ± 0.30	2.32 ± 0.41	0.867
Change	−0.02 ± 0.22	−0.01 ± 0.19	0.811
Intragroup p value	0.657	0.890	

Data are presented as the mean ± SD (n = 21 for both groups). p values < 0.05 indicate significant differences. Comparisons were performed by one-sample *t*-test in each group, and two-sample *t*-tests between groups. SRS: summed rest score, H/M: heart-to-mediastinal ratio. *p < 0.05

### Physical and metabolic parameters

Body weight was significantly reduced only in the empagliflozin group from baseline to 12 weeks, and no significant difference was observed in body weight reduction between the groups (Table 4). Additionally, HbA1c was significantly decreased to a similar extent in both groups (Table 4). Meanwhile, plasma glucose, plasma insulin, and HOMA-IR were significantly lower in the empagliflozin group than in the sitagliptin group (Table 4). High-density lipoprotein (HDL) cholesterol and apolipoprotein A-I were significantly higher in the empagliflozin group than in the sitagliptin group, whereas low-density lipoprotein cholesterol and apolipoprotein B were similar between the groups (Table 4). Uric acid was significantly decreased in the empagliflozin group compared to the sitagliptin group (Table 4). Total ketone bodies, β-hydroxybutyric acid, and acetoacetic acid were significantly higher in the empagliflozin group than in the sitagliptin group (Table 4). Although the hematocrit level was significantly increased in the empagliflozin group, no significant differences were observed between the groups (Additional file 1). Blood pressure, brain natriuretic peptide (BNP), and heart-type fatty acid-binding protein were not statistically different between the groups (Additional file 1).

**Table 4:** Physical and metabolic parameters

	Empagliflozin	Sitagliptin	p value
Body weight (kg)			
Baseline	80.3 ± 19.0	84.4 ± 16.1	0.469
12 weeks	79.1 ± 19.6	83.7 ± 16.3	0.411
Change	−1.2 ± 1.6	−0.2 ± 2.0	0.081
Intragroup p value	0.002*	0.679	
HbA1c (%)			
Baseline	7.1 ± 0.8	7.0 ± 0.9	0.684
12 weeks	6.7 ± 0.6	6.6 ± 0.8	0.895
Change	−0.5 ± 0.4	−0.4 ± 0.4	0.549
Intragroup p value	< 0.001*	< 0.001*	
Plasma glucose (mg/dL)			
Baseline	155.7 ± 34.2	140.6 ± 33.3	0.155
12 weeks	124.6 ± 18.2	135.3 ± 37.2	0.243
Change	−31.1 ± 21.8	−5.3 ± 18.3	< 0.001*
Intragroup p value	< 0.001*	0.200	
Plasma insulin (µIU/mL)			
Baseline	13.7 ± 7.1	14.4 ± 9.5	0.795
12 weeks	9.4 ± 5.5	14.9 ± 7.3	0.009*
Change	−4.4 ± 6.4	0.5 ± 6.5	0.019*
Intragroup p value	0.005*	0.735	
HOMA-IR			
Baseline	5.3 ± 2.8	4.9 ± 3.7	0.718
12 weeks	2.9 ± 1.8	4.8 ± 2.8	0.011*
Change	−2.4 ± 2.4	−0.1 ± 2.5	0.004*
Intragroup p value	< 0.001*	0.902	
HDL cholesterol (mg/dL)			
Baseline	52.4 ± 14.4	48.8 ± 8.5	0.333
12 weeks	54.0 ± 14.7	46.5 ± 10.1	0.061
Change	1.7 ± 6.3	−2.3 ± 5.3	0.034*
Intragroup p value	0.241	0.061	
Apolipoprotein A-I (mg/dL)			
Baseline	146.7 ± 30.4	145.2 ± 22.8	0.860
12 weeks	147.3 ± 30.5	138.1 ± 22.7	0.277
Change	0.6 ± 12.2	−7.1 ± 10.6	0.036*
Intragroup p value	0.833	0.006*	
LDL cholesterol (mg/dL)			
Baseline	134.4 ± 29.7	139.2 ± 39.7	0.659
12 weeks	132.8 ± 22.7	135.0 ± 41.0	0.824
Change	−1.7 ± 16.3	−4.2 ± 17.9	0.636
Intragroup p value	0.644	0.297	
Apolipoprotein B (mg/dL)			
Baseline	110.1 ± 25.1	113.0 ± 24.3	0.715
12 weeks	107.5 ± 22.0	109.3 ± 25.9	0.808
Change	−2.6 ± 10.9	−3.6 ± 12.4	0.783
Intragroup p value	0.285	0.197	
Uric acid (mg/dL)			
Baseline	5.8 ± 1.2	6.3 ± 1.2	0.171
12 weeks	5.0 ± 0.9	6.5 ± 1.2	< 0.001*
Change	−0.8 ± 0.9	0.1 ± 0.8	< 0.001*
Intragroup p value	< 0.001*	0.446	
Total ketone bodies (μmol/L)			
Baseline	81.8 ± 50.3	107.0 ± 82.6	0.725
12 weeks	233.6 ± 372.3	93.5 ± 58.0	0.138
Change	151.8 ± 335.2	−13.5 ± 59.5	0.002*
Intragroup p value	< 0.001*	0.591	
β-hydroxybutyric acid (μmol/L)			
Baseline	52.9 ± 33.7	72.2 ± 59.7	0.624
12 weeks	159.6 ± 263.3	60.0 ± 38.9	0.099
Change	106.7 ± 239.2	−12.2 ± 42.4	0.001*
Intragroup p value	< 0.001*	0.370	
Acetoacetic acid (μmol/L)			
Baseline	28.9 ± 16.9	34.8 ± 23.6	0.357
12 weeks	74.0 ± 109.3	33.5 ± 19.8	0.102
Change	45.1 ± 96.3	−1.3 ± 18.0	0.036*
Intragroup p value	0.044*	0.746	

Data are presented as the mean ± SD (n = 21 for both groups). p values < 0.05 indicate significant differences. Comparisons were performed by one-sample *t*-test in each group, and two-sample *t*-tests between groups. HDL: high-density lipoprotein, HOMA-IR: homeostasis model assessment of insulin resistance, LDL: low-density lipoprotein. *p < 0.05

### Safety outcomes

The total number of AEs was significantly higher in the empagliflozin group than in the sitagliptin group (Additional file 2). This was primarily due to increased urination, a common pharmacological effect of SGLT2 inhibitors, in the empagliflozin group [5 (23.8%) vs. 0 (0.0%), p < 0.05]. No patient dropped out of the study due to AEs associated with drug administration.

## Discussion

This study demonstrated that 12-week administration of empagliflozin had similar effects as sitagliptin on cardiac fat accumulation and cardiac function in patients with early-stage T2DM, without CVD complications. However, certain cardiometabolic biomarkers were significantly improved in the empagliflozin group compared to those in the sitagliptin group. These changes in cardiometabolic biomarkers by early empagliflozin supplementation may contribute to the primary prevention of CVD.

It has been shown that SGLT2 inhibitors, such as luseogliflozin, ipragliflozin, and canagliflozin significantly reduced epicardial fat accumulation in a 12-week one-arm study [18–20]. It was also shown that the reduction with dapagliflozin was larger than that with conventional therapy [21]. However, no changes in pericardial, epicardial, or paracardial fat accumulation following 12 weeks of empagliflozin treatment were assessed in these studies. Moreover, considering that the previous studies were one-arm or placebo-controlled trials in patients with various backgrounds, and the methods for cardiac fat evaluation varied, it is difficult to directly compare the previous results with ours. Nevertheless, patients in the current study were relatively younger (average 52.8 years) with lower HbA1c levels (average 7.1%) and shorter diabetes durations (average 3.5 years) than those reported in the previous studies (mean age range: 55–68 years; mean HbA1c range, 7.1–7.5%) [18–21]. Furthermore, patients had few microvascular and macrovascular complications in this study (only one subject had diabetic nephropathy and another had peripheral arterial disease). Therefore, these conflicting results might have originated from differences in baseline characteristics, such as the severity of T2DM or the existence and extent of vascular complications. In addition, the method employed for determination of cardiac fat accumulation differed between the current study and the previous studies. Specifically, our study used cine MRI to evaluate epicardial and pericardial fat and myocardial triglyceride content, which has been reported previously by another group [36], but the other studies used whole-heart coronary magnetic resonance angiography [18, 19], echocardiography [20, 22], or cardiac computed tomography [21] to evaluate epicardial fat content. Such differences in evaluation methods may also account for the different results in cardiac fat accumulation between the present and previous studies. Moreover, myocardial triglyceride content was unaffected despite its baseline value being higher in our subjects than in healthy Japanese subjects [37] or patients with left ventricular hypertrophy [38]. This result is consistent with that of a previous one-arm study showing that 6 months of empagliflozin treatment did not impact myocardial triglyceride content [45]. Considering that no clinical studies have assessed the effect of DPP-4 inhibitors on myocardial triglyceride content or compared the effects of SGLT2 inhibitors and DPP-4 inhibitors on cardiac lipid levels, this is the first study to show that SGLT2 inhibitors and DPP-4 inhibitors do not affect pericardial fat accumulation or myocardial triglyceride content at the early-stage of T2DM. Therefore, further studies are necessary to establish the most effective approaches for reducing pericardial and epicardial fat accumulation and myocardial triglyceride content and to clarify the impact of these reductions on CVD prevention.

Additionally, no differences were noted in the effects on cardiac function and mass between the empagliflozin and sitagliptin groups. This is consistent with a previous study showing that dapagliflozin did not alter cardiac function after 12 weeks of treatment [46]. However, other studies enrolling patients with T2DM and chronic heart failure or coronary heart disease demonstrated that 24 weeks of dapagliflozin and empagliflozin treatment improved the ratio of mitral inflow E to mitral e′ annular velocities, an indicator of diastolic function, while reducing left ventricular mass [47, 48]. Considering these previous studies, 12 weeks might have been insufficient to improve cardiac function and structure; furthermore, since cardiac function and structure were well-preserved at baseline in this study, no further improvement may have been detectable following empagliflozin treatment. In addition, a recent study reported that pericardial fat volume was associated with diastolic function even in healthy subjects with normal cardiac function [16], which supports our results showing that no reduction in pericardial fat volume or improvement in diastolic function was observed in parallel. Interestingly, although there was no difference in cardiac function between the groups, sitagliptin significantly decreased LVEF and  %FS from baseline to 12 weeks. Similarly, Mulvihill et al. demonstrated that a DPP-4 inhibitor impaired cardiac function in a rodent model [49] and Li et al. reported that DPP-4 activity was positively correlated with LV systolic function, suggesting that DPP-4 inhibition is correlated with LV systolic dysfunction in humans [50]. Furthermore, various CVD outcome trials have indicated that DPP-4 inhibitors increase the risk of hospitalizations due to heart failure [9, 51]. Unexpectedly, SRS, as assessed by ^123^I-BMIPP scintigraphy, which indicates myocardial fatty acid uptake and is associated with LV wall motion abnormality [27] as well as the incidence of CVD events [28, 52], showed significantly decreased values from baseline only in the sitagliptin group, although the values were not statistically different between groups. This reduction may be due to the higher baseline values in the sitagliptin group and, thus, may not have clinical implications considering that the patients in the sitagliptin group had no history of CVD events. Indeed, normal cardiac function was observed by echocardiography, which is a common and reliable diagnostic method.

In addition to direct evaluation of the heart, we measured cardiometabolic indices. Although no significant differences were observed in body weights or HbA1c levels between the groups, plasma glucose, plasma insulin, and HOMA-IR were significantly decreased from baseline in the empagliflozin group compared to the sitagliptin group values, indicating that empagliflozin can improve insulin resistance, an independent risk factor for CVD [53, 54]. In addition, empagliflozin preserved the serum levels of HDL cholesterol and apolipoprotein A-I, the latter of which is a component of HDL cholesterol, whereas it was significantly decreased in the sitagliptin group. A previous study reported that the risk of myocardial infarction was increased by approximately 25% for every 5 mg/dL decrease in the serum HDL cholesterol level [55]. The uric acid level, which positively associates with CVD risk through hypertension and vascular damage, was also significantly decreased in the empagliflozin group [56, 57]. Meanwhile, the levels of total ketone bodies, β-hydroxybutyric acid, and acetoacetic acid were markedly higher in the empagliflozin group than in the sitagliptin group. Ketone bodies serve as an energy source for the heart along with glucose and free fatty acids. Particularly, β-hydroxybutyric acid is thought to be a “super fuel” for the heart, as infusion of β-hydroxybutyric acid was shown to improve cardiac function and structural remodeling in rodent models [58, 59]. Therefore, increased ketone bodies may have a protective cardiac function in the empagliflozin group. Although no decrease was observed in BNP or heart-type fatty acid-binding protein levels, this may have been due to normally low levels at baseline, similar to that discussed above for the cardiac parameters. Supporting this, Soga et al. reported that an SGLT2 inhibitor improved BNP only in patients with BNP ≥ 100 pg/mL [60]. Taken together, these results suggest that, compared to sitagliptin, empagliflozin plays a greater role in preventing future CVD in the early stage of diabetes, without CVD complications.

Previously, we reported that linagliptin, a DPP-4 inhibitor, and dapagliflozin both protect endothelial function in patients with early-stage T2DM [61, 62]. In addition, we reported that dapagliflozin was more effective than sitagliptin for lowering HbA1c levels, reducing body weight, and avoiding hypoglycemia in early-stage T2DM, which may lead to CVD prevention [63]. However, the current study is the first to compare the cardiovascular and cardiometabolic effects of empagliflozin and sitagliptin in a randomized controlled trial in patients with early-stage diabetes with no complications of CVD and with preserved cardiac function. Although the direct effects on cardiac lipid accumulation and cardiac function did not differ between the two groups, empagliflozin was superior to sitagliptin for cardiometabolic parameters, such as uric acid, HDL cholesterol, ketone bodies, and insulin sensitivity following only 12 weeks of treatment, which was consistent with a previous report [64]. Further studies are needed to clarify how these results affect long-term cardioprotection; however, early administration of SGLT2 inhibitors would be beneficial for the primary prevention of CVD, as well as secondary prevention.

This study had the following limitations. First, the small sample size and short observation period may partially explain the lack of significant differences in cardiac parameters. Second, patients had a short history of diabetes and no CVD, which may have made it difficult to detect significant differences in cardiac parameters between the groups. Finally, while metformin is highly recommended as the first-line drug in Europe and the United States [65], the use of metformin was avoided in this study to eliminate its effects on insulin sensitivity and cardiac protection in patients who were overweight [66]. Interestingly, it was reported that metformin may moderate CVD outcomes with DPP-4 inhibitor use [67] but SGLT2 inhibitors provide cardioprotective effects regardless of concomitant metformin use [68]. In this regard, this study is the first to compare the cardioprotective effects directly between SGLT2 inhibitors and DPP-4 inhibitors without metformin use in patients with early-stage diabetes without CVD that includes heart failure. Overall, although we comprehensively compared the cardioprotective effects of SGLT2 inhibitors and DPP-4 inhibitors in a randomized controlled trial, clinical studies of a large sample size with subjects of various ethnicities are warranted to confirm our results.

## Conclusions

No significant differences were observed in the effects on cardiac fat accumulation, cardiac function, and cardiac fatty acid metabolism between the empagliflozin and sitagliptin groups after 12 weeks of treatment. However, regarding cardiometabolic biomarkers, empagliflozin significantly decreased serum uric acid and increased HDL cholesterol, ketone bodies, and insulin sensitivity compared to the corresponding sitagliptin values. Therefore, early supplementation with SGLT2 inhibitors may be preferable to DPP-4 inhibitors to provide early cardiac protection and primary prevention of CVD in patients with early-stage T2DM and preserved cardiac function.

## Supplementary information

**Additional file 1.** Additional patient parameters (physical and biochemical parameters that showed no significant differences between the empagliflozin and sitagliptin groups).

**Additional file 2.** Adverse events (list of observed adverse events).

## Data Availability

The datasets analyzed during the current study are available from the corresponding author upon reasonable request.

## Abbreviations

AE: Adverse event
BNP: Brain natriuretic peptide
CVD: Cardiovascular disease
DPP-4: Dipeptidyl peptidase-4
FAS: Full analysis set
HbA1c: Glycated hemoglobin
HDL: High-density lipoprotein
HOMA-IR: Homeostasis model assessment-insulin resistance
LV: Left ventricular
LVEF: Left ventricle ejection fraction
MRI: Magnetic resonance imaging
SGLT2: Sodium-glucose cotransporter-2
SPECT: Single-photon emission computed tomography
SRS: Summed rest score
T2DM: Type 2 diabetes mellitus
^1^H-MRS: Proton magnetic resonance spectroscopy
^123^I-BMIPP scintigraphy: Iodine-123-β-methyl-iodophenyl pentadecanoic acid myocardial scintigraphy
%FS: Percent fractional shortening
