# Supplementary material for: A prospective randomized study comparing effects of empagliflozin to sitagliptin on cardiac fat accumulation, cardiac function, and cardiac metabolism in patients with early-stage type 2 diabetes: the ASSET study
Source: Cardiovasc Diabetol. 2021 Feb 2;20:32. doi: 10.1186/s12933-021-01228-3 (PMC7852076; doi:10.1186/s12933-021-01228-3)
Supplement: Supplementary file 1 — Additional file 1. Additional patient parameters (physical and biochemical parameters that showed no significant differences between the empagliflozin and sitagliptin groups). [file 12933_2021_1228_MOESM1_ESM.docx]

**Additional file 1. Additional patient parameters**

|  | Empagliflozin | Sitagliptin | p value |
| --- | --- | --- | --- |
| Systolic blood pressure (mmHg) |  |  |  |
| Baseline | 140.8 ± 21.1 | 146.4 ± 26.3 | 0.465 |
| 12 weeks | 135.7 ± 15.2 | 137.0 ± 17.5 | 0.814 |
| Change | -6.1 ± 16.1 | -7.8 ± 19.8 | 0.777 |
| Intragroup p value | 0.141 | 0.122 |  |
| Diastolic blood pressure (mmHg) |  |  |  |
| Baseline | 88.9 ± 11.1 | 87.7 ± 12.6 | 0.760 |
| 12 weeks | 85.7 ± 10.0 | 84.7 ± 9.5 | 0.748 |
| Change | -4.0 ± 9.5 | -3.0 ± 11.2 | 0.780 |
| Intragroup p value | 0.102 | 0.284 |  |
| Hematocrit (%) |  |  |  |
| Baseline | 43.2 ± 3.8 | 44.1 ± 2.9 | 0.387 |
| 12 weeks | 44.8 ± 3.5 | 44.7 ± 3.3 | 0.903 |
| Change | 1.6 ± 2.0 | 0.6 ± 2.3 | 0.128 |
| Intragroup p value | 0.002* | 0.256 |  |
| Triglyceride (mg/dL) |  |  |  |
| Baseline | 184.3 ± 166.1 | 186.3 ± 137.7 | 0.966 |
| 12 weeks | 166.4 ± 137.3 | 183.6 ± 182.8 | 0.732 |
| Change | -17.9 ± 67.0 | -2.7 ± 69.3 | 0.476 |
| Intragroup p value | 0.236 | 0.859 |  |
| BNP (pg/mL) |  |  |  |
| Baseline | 8.9 ± 5.8 | 13.5 ± 12.8 | 0.138 |
| 12 weeks | 8.7 ± 5.3 | 8.3 ± 5.1 | 0.784 |
| Change | -0.2 ± 5.8 | -5.2 ± 11.9 | 0.086 |
| Intragroup p value | 0.897 | 0.056 |  |
| H-FABP (μEq/L) |  |  |  |
| Baseline | 4.15 ± 2.84 | 4.15 ± 2.40 | 0.995 |
| 12 weeks | 4.22 ± 2.62 | 4.07 ± 2.38 | 0.845 |
| Change | 0.07 ± 0.64 | -0.08 ± 1.02 | 0.577 |
| Intragroup p value | 0.612 | 0.736 |  |

Data are presented as the mean ± SD (n = 21 for both groups). p values < 0.05 indicate significant differences. Comparisons were performed by one-sample *t*-test in each group, and two sample *t*-tests between groups. BNP brain natriuretic peptide, H-FABP heart type fatty acid-binding protein. * p < 0.05.
