# Supplementary material for: A prospective randomized study comparing effects of empagliflozin to sitagliptin on cardiac fat accumulation, cardiac function, and cardiac metabolism in patients with early-stage type 2 diabetes: the ASSET study
Source: Cardiovasc Diabetol. 2021 Feb 2;20:32. doi: 10.1186/s12933-021-01228-3 (PMC7852076; doi:10.1186/s12933-021-01228-3)
Supplement: Supplementary file 2 — Additional file 2. Adverse events (list of observed adverse events). [file 12933_2021_1228_MOESM2_ESM.docx]

**Additional file 2. Adverse events**

|  | **Empagliflozin** | **Sitagliptin** | **p Value** |
| --- | --- | --- | --- |
| Any adverse events | 12 (57.1) | 5 (21.7) | 0.029* |
| Back pain | 0 (0.0) | 1 (4.3) | 1.000 |
| Constipation | 1 (4.8) | 0 (0.0) | 0.477 |
| Dysphoria | 0 (0.0) | 1 (4.3) | 1.000 |
| Frequent urination | 5 (23.8) | 0 (0.0) | 0.019* |
| Floating vertigo | 1 (4.8) | 0 (0.0) | 0.477 |
| Heat stroke | 0 (0.0) | 1 (4.3) | 1.000 |
| Onychomycosis | 1 (4.8) | 0 (0.0) | 0.477 |
| Palpitation | 1 (4.8) | 0 (0.0) | 0.477 |
| Pharyngitis | 1 (4.8) | 1 (4.3) | 1.000 |
| Seasonal allergy | 1 (4.8) | 0 (0.0) | 0.477 |
| Abdominal pain | 1 (4.8) | 0 (0.0) | 0.477 |
| Thirst | 3 (14.3) | 0 (0.0) | 0.100 |
| Upper respiratory tract infection | 1 (4.8) | 0 (0.0) | 0.477 |
| Urticaria | 0 (0.0) | 1 (4.3) | 1.000 |

N = 21 and 23 for empagliflozin group and sitagliptin group, respectively. Data were acquired from the safety analysis set. p values < 0.05 indicate significant differences. Comparisons were performed using Fisher’s exact test. * p < 0.05.
